# The Biological and Mechanical Effect of Using Different Irrigation Methods on the Bond Strength of Bioceramic Sealer to Root Dentin Walls

**DOI:** 10.7759/cureus.24022

**Published:** 2022-04-11

**Authors:** Dania F Bogari, Mohammed Alessa, Mahmoud Aljaber, Faisal Alghamdi, Mohammed Alamoudi, Mohammed Alhamed, Abdulrahman J Alghamdi, Samia Elsherief, Majed Almalki, Turki Y Alhazzazi

**Affiliations:** 1 Endodontics, Faculty of Dentistry, King Abdulaziz University, Jeddah, SAU; 2 Restorative Dentistry, Alhada Armed Hospital, Taif, SAU; 3 Endodontics, King Salman Armed Hospital, Tabuk, SAU; 4 Oral Biology, Faculty of Dentistry, King Abdulaziz University, Jeddah, SAU; 5 General Dentistry, Ministry of Health, Taif, SAU; 6 General Dentistry, Ministry of Health, Makkah, SAU; 7 Restorative Dentistry, Faculty of Dentistry, Umm Al-Qura University, Makkah, SAU

**Keywords:** bond strength, ultrasonic, laser, irrigation, bioceramic sealer

## Abstract

Objectives

This study aimed to evaluate the biological and mechanical effect of different irrigation methods on the bond strength of Bioceramic (BC) sealer to root canal dentin walls.

Material and Methods

Forty-Five single-rooted teeth were decoronated and then prepared using rotary instrumentation. Teeth were randomly divided into three groups. Group 1: using the conventional syringe method; Group 2: using the ultrasonic (US) activation method; and Group 3: using the Nd:YAG laser activation method. The BC sealer (TotalFill^®^ BC Sealer™, FKG Dentaire, Switzerland) was used for obturation according to the manufacturer’s recommendation. The bond strength was evaluated using the push-out test, and the adaptation of the sealer/dentin interface was assessed using Scanning Electron Microscope (SEM). Data were analyzed by Welch’s ANOVA analysis of variance and Games-Howell for pairwise comparison. The level of statistical significance was set at 95% (*p*-value ≤ 0.05).

Results

The push-out bond strength values of the Nd:YAG (6.46 ± 0.5) laser group were statistically significant than both conventional (3.33 ± 1.8) and US groups (4.21 ± 2.2). The mean gaps that were formed between the root walls and GP/BS sealer interface were statistically significant only between the Nd:YAG laser group (25.54 ± 13.8) compared to both conventional (62.00 ± 15.3) and US groups (58.82 ± 23.8) (*p *≤0.05). No significant difference was found between the conventional and US groups in both rested parameters (*p* >0.05).

Conclusion

The method protocol of RC system irrigation affects the adhesion and bond strength of BS sealers to the root canal dentin walls.

## Introduction

Successful root canal treatment depends on proper canal debridement, elimination of pathogenic organisms, and finally sealing the root canal system [1-3]. As gutta-percha (GP) does not bind directly to the root dentin walls, the sealer should fill the gaps between the GP core material and root dentin walls to ensure a proper seal of all the root canal systems [4].

Bioceramic (BC) sealers seem to have a favorable sealing ability, biocompatibility and exhibit favorable characteristics with promising treatment outcomes compared to other root canals sealers [5,6]. Studies demonstrated that BC sealers have dimensional stability because they are highly alkaline (pH ≥12), and they do not shrink upon setting [7,8].

The conventional irrigation method of using a syringe loaded with an irrigation solution is commonly used by general practitioners and endodontists [9,10]. The syringe needle is embedded close to the working length, and the irrigation solution is conveyed by which it streams through the canal orifice [11]. It is also known as positive pressure irrigation as it creates a pocket of pressure in the apical third of the root canal. Sodium hypochlorite (NaOCl) and ethylenediamine tetra-acetic acid (EDTA) are the most commonly employed root canal irrigation solutions in dental practice [12]. NaOCl is used to remove the organic component of the smear layer, whereas EDTA is used to remove the inorganic component of the smear layer after root canal (RC) system debridement [13]. Unfortunately, the conventional method using these irrigants cannot eliminate root canals debris effectively [14]. Thus, multiple activation methods were warranted to effectively remove microorganisms, pulp tissue, and smear layer from the root canal system. Examples of those are passive ultrasonic irrigation (PUI) and laser-activated irrigation (LAI) [15-20]. However, the type of irrigation solution and methods used for RC system irrigation may affect the quality of adhesion between root canal dentin and newly developed sealers, such as the BC sealer.

Few studies have investigated the degree of bonding and adaptation of different sealers to root canal dentin under different RC treatment conditions [21-25]. However, to our knowledge, no study has determined the effect of different irrigation methods including conventional, ultrasonic, and laser activation irrigation, on the sealer-dentin bond strength when using bioceramics (BC) as a root canal treatment sealer. Thus, our study aimed to evaluate the biological and mechanical effect of different irrigation methods, including conventional, ultrasonic, and laser activation irrigation, on the bond strength of BC sealer to root canal dentin. The null hypothesis stated that there is no significant difference in the bond strength of BS sealer to root canal walls regarding the irrigation methods used.

## Materials and methods

This research was approved by the ethical research committee review board at Umm Al-Qura University, Faculty of Dentistry, Makkah, Saudi Arabia with (Ref.: #117-18), and was in full accordance with the World Medical Association Declaration of Helsinki.

Sample selection

Forty-five freshly extracted single-root human teeth with fully developed and straight roots obtained from different private and governmental oral surgery clinics in Makkah, Saudi Arabia were selected for this study. These roots were confirmed to be radiographically free from open apices, restorations, caries, cracks, or fractures. Selected teeth were then cleaned and stored in a 10% formalin solution at room temperature.

Sample grouping

In this *in vitro* study, samples were randomly divided into three groups (15 teeth per group) according to the type of irrigating method used for disinfecting the root canal system: Group 1: Conventional irrigation method (n = 15); Group 2: Ultrasonic activation irrigation method (n = 15); and Group 3: Laser activation irrigation method (n = 15).

Each of the three groups was then divided into two subgroups (n = 7 and n = 8) to assess the bond strength, and to evaluate the studied subgroups under a scanning electron microscope (SEM), respectively.

Sample preparation

For all 45 selected teeth, the coronal part of the tooth was removed at a level below the cementoenamel junction (CEJ) to prepare a standardized root length of 15 mm, using a diamond disc with water coolant. The canal patency was verified in all canals samples by inserting an initial file size #15 K-file into the RC, then advancing the file until visualized at the apical foramen. The working length was established using size #15 K-file 1 mm short of the apex. The preparation of all RC was completed using Pro Taper Next files (Dentsply Maillefer, Ballaigues, Switzerland) up to X3 file according to the manufacturer’s instruction. During root canals preparation, the following irrigation methods were used according to the corresponding studied groups: 

Group 1: Conventional Irrigation Method

During the cleaning and shaping of the root canal system, canals were irrigated via the conventional irrigation method using 2 ml of 5.25% sodium hypochlorite (NaOCl) for 120 seconds between each file via 5 ml syringe/gauge 30 needles. The needle was placed 1 mm from working length and moved in an up-and-down motion.

Group 2: Ultrasonic Activation Irrigation Method

During the cleaning and shaping of the root canal system, the irrigation solution was activated using the ultrasonic device for 1 min by an ultrasonic tip adaptor (Helse Ultrasonic, Brazil) holding a noncutting stainless steel wire size #20. The ultrasonic device (Suprasson Pmax Newtron, Satelec, Acteongroup) was set at the power of 4. The 15 samples were irrigated with 2 ml of 5.25% NaOCl for 120 seconds. The ultrasonic tip was placed at a 1-mm distance from the working length and moved up and down motion.

Group 3: Laser Activation Irrigation Method

During the cleaning and shaping of the root canal system, the Nd: YAG laser (LaserHF® “comfort”, Duisburg, Germany) was used for irrigation activation with a panel setting of 1.6 W (Power), 25 Hz (Frequency), 975 nm wavelength, and energy of 100 mJ with a short pulse of 200 μs. The fiber tip was fixed in the handpiece of the Nd: YAG laser, then 2 ml of 5.25% NaOCl was placed into the RC, and the optical fiber was placed at 3 mm from the root apex and pulled out slowly within 10-30 seconds with a circular movement. During the laser irradiation, continuous irrigation was maintained to ensure hydration. Each root canal of the 15 samples in this group was irrigated for 120 seconds.

The final irrigation was performed using 17% ethylenediaminetetraacetic acid (EDTA) solutions left inside the canal for 1 min to ensure complete removal of the smear layer, followed by 10 ml of distilled water to remove any remaining chemicals and debris within the RC system [26]. All canals were dried using a vacuum adaptor tip using low suction for 5 seconds, and flowed by one single paper point for 1 second [26]. The BC sealer (TotalFill® BC Sealer™, FKG Dentaire, Switzerland) was injected inside the canals through disposable tips up to the coronal portion of the canal and obturated by using single cone gutta-percha (GP) according to the manufacturer’s recommendations. The excess GP was then removed and compressed using a hand plugger. All samples were wrapped in a wet gauze and then stored in closed plastic containers at 37°C with 100% humidity for 48 hours, allowing the sealer to set completely. All samples were stored for 2 weeks until the bond strength test was performed and samples were then scanned under SEM.

Bond Strength Testing

**Figure 1:**
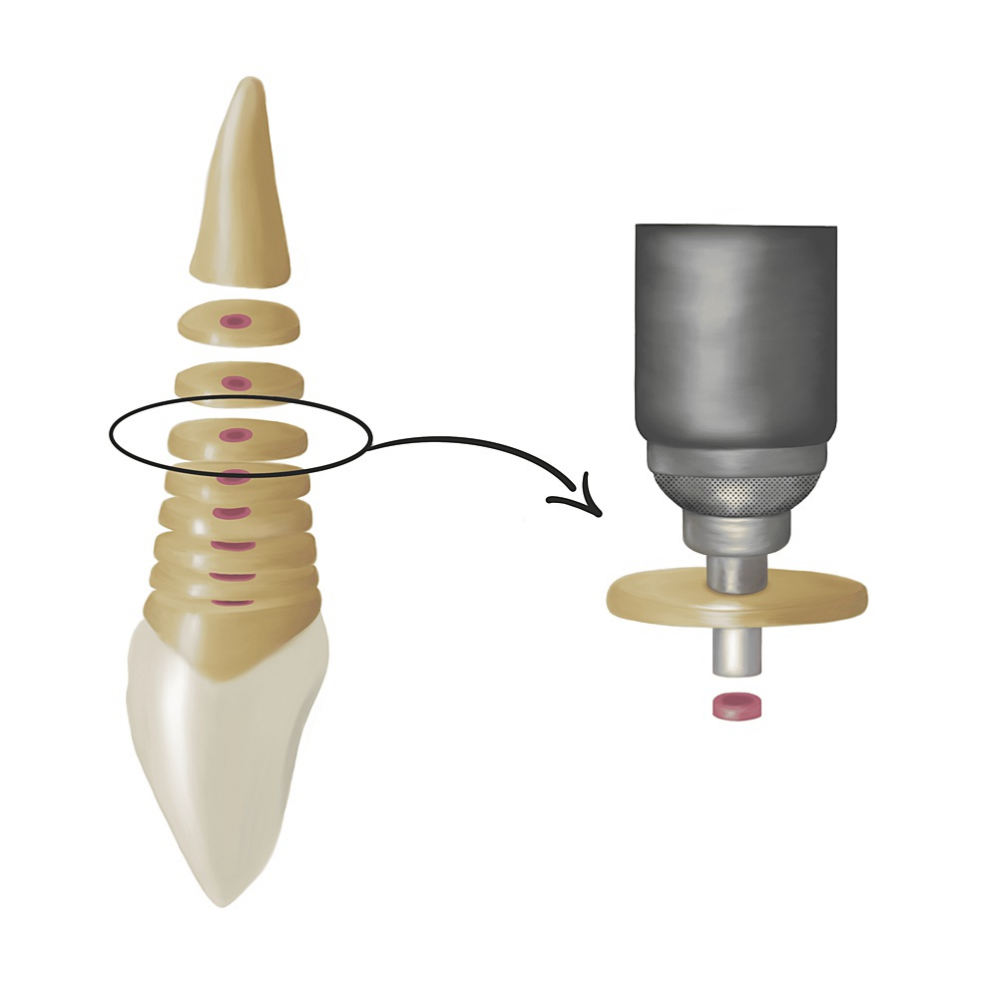
Schematic representation of sample tooth preparation and the push-out setup test used for bond strength evaluation.

Samples were horizontally sectioned into 2 mm lengths using a diamond disk (Figure 1). All 45 teeth were mounted vertically in self-cured acrylic resin mounting blocks (1.5 × 1.5 mm). Teeth embedded in the acrylic blocks were placed in a universal testing machine (3400 SERIES, Instron®, MA, USA) to determine the bond strength using the push-out technique. This was accomplished by using a 0.5 mm diameter stainless steel plunger. A constant compressive load was applied until bond failure occurred at 1 mm/min (Figure 1). The bond strength was recorded in megapascals (MPa), as previously described by Skidmore et al. [27].

Scanning Electron Microscope (SEM) Analysis

The sectioned samples were dehydrated for assessment under SEM (Inspect S50 model, FEI™, Japan). Following mounting, samples were coated with a thin gold layer with a coater system (Quorum). Under SEM, two representative areas from the middle and apical third of each sample were focused. Then, all specimens were prepared using the SEM and photographed at 400× magnifications. The device system of SEM was adjusted to operate at a high vacuum [3.0 nm at 30 kV scanning electron (SE)]. The Image Tool Pro Plus 6.0 software (Media Cybernetics, USA) was used to measure the adaptation of the BS sealer at the sealer/dentin interface by analyzing the gaps between the BS sealer and the root dentin walls at 2 mm from the apex of the tooth, measured in microns (μm) among all three irrigation methods groups.

Statistical analysis 

The push-out bond strength (MPa) data were analyzed using Welch’s ANOVA Test between the three irrigation methods groups. Test of Homogeneity of Variances (Levene Statistic) was used as a normality test to check if the samples have equal variances. For pairwise comparisons, the Games-Howell test was used. The complete computational work was performed with the aid of IBM SPSS version 23 (IBM Corp., Armonk, N.Y., USA) and visually presented using GraphPad Prism version 8 (GraphPad Software, Inc., San Diego, CA, USA). The level of statistical significance was set at 95% (p ≤ 0.05).

## Results

**Figure 2:**
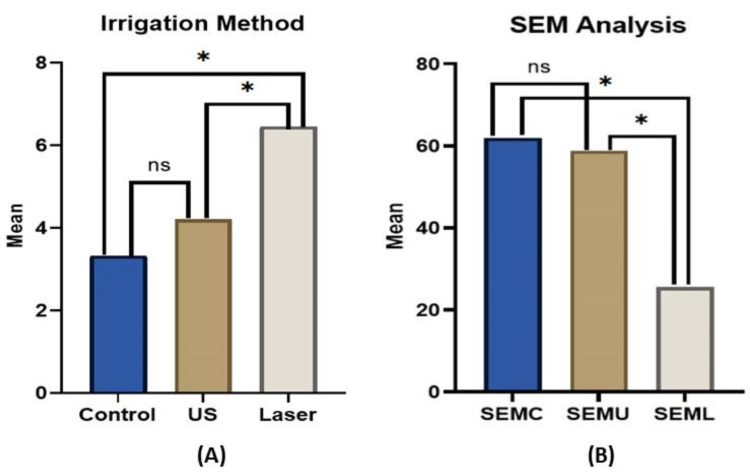
(A) Comparison of push-out bond strength mean values (Mpa). The Nd:YAG laser group was significantly stronger than the conventional and US groups (p ≤0.05). However, the difference between the conventional and US groups was not significant (p >0.05). (B) SEM gap mean values between all tested groups. The mean gaps that formed between the root wall and GP/BS sealer interface were significantly different only in the Nd:YAG laser group compared to both conventional and US groups (p ≤0.05). No significant difference was found between the conventional and US groups in both rested parameters (p >0.05). ^* ^significant difference. ^ns^ no significant difference. (US): Ultrasonic. (SEMC): Scanning Electron Microscope - Conventional group. (SEMU): Scanning Electron Microscope - Ultrasonic group. (SEML): Scanning Electron Microscope - Laser group.

**Figure 3:**
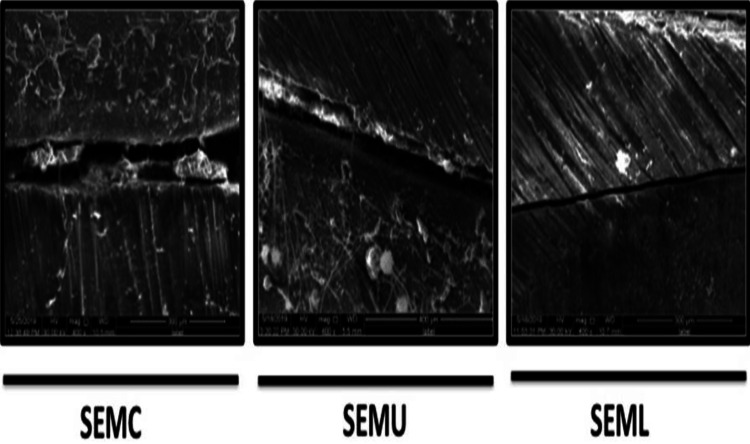
Representative scanning electron microscope showing the gap differences between root dentinal walls and GP/BS sealer interface among all tested groups. Conventional syringe irrigation (SEMC), Ultrasonic activation irrigation (SEMU), and Nd:YAG laser activation (SEML). (SEMC): Scanning Electron Microscope - Conventional group. (SEMU): Scanning Electron Microscope - Ultrasonic group. (SEML): Scanning Electron Microscope - Laser group.

The comparison of push-out bond strength mean values (Mpa) and SEM gap mean values between all tested groups is presented in Table 1. The BS sealer’s push-out bond strength and SEM values were significantly affected by the type of irrigation method used (p ≤0.05). Thus, our results reveal that the mean push-out bond strength values of the Nd:YAG (6.46 ± 0.5) laser group were higher than both the conventional (3.33 ± 1.8) and US (4.21 ± 2.2) groups (Table 1). However, the difference between the conventional and US groups was not significant (p >0.05) (Table 1 and Figure 2). On the other hand, regarding SEM analysis, gap differences between the root walls and GP/BS sealer interface were recorded between all tested groups (Table 1 and Figure 2B). Thus, the mean gaps that were formed between the root walls and GP/BS sealer interface were as follows: laser groups (~25 μm), ultrasonic groups (~58 μm), and conventional groups (~61 μm) (Table 1 and Figure 2B). However, this was statistically significant only between the laser group compared to both conventional and US groups (p ≤0.05) (Table 1 and Figure 2B). No significant difference was found between the conventional and US groups in both rested parameters (p >0.05). A representative SEM image showing the gap differences between dentinal root walls and GP/BS sealer interface is represented in Figure 3.

**Table 1:** Comparison of three tested groups with respect to mean push-out bond strength (Mpa) and gap width between groups. ^a-^significant using Welch's ANOVA Test at <0.05 level. ^b-^Post Hoc Test = Games-Howell. (SD): standard deviation.

Irrigation Method ^b^	Bond Strength	SEM
	Mean ± SD	Mean ± SD
Conventional Group	3.33 ± 1.8	62.00 ± 15.3
Ultrasonic Group	4.21 ± 2.2	58.82 ± 23.8
Laser Group	6.46 ± 0.5	25.54 ± 13.8
p-value	<0.001^a^	<0.001^a^

## Discussion

Our results reveal that the type of irrigation method affects the bond strength of the BC sealer to root canals walls. Thus, the null hypothesis is rejected. Irrigation by the laser activation method resulted in significantly higher bonding strength and fewer gaps of BS sealer to the root-dentin interface compared to the US and conventional irrigation methods. To our knowledge, our study is the first to study the effect of three different irrigation methods: conventional syringe method, ultrasonic (US) activation method, and Nd:YAG laser activation method, while using 5.25% NaOCL as the main irrigation solution on the bond strength of BS sealer to root canals walls system. This study used the traditional irrigation protocol of 5.25% NaOCL followed by 17% EDTA to remove the organic and inorganic smear layer, respectively [13]. As the effect of NaOCL and other commonly used irrigation solutions on the bond strength of different sealers types including BS sealers, is still controversial, distilled water was used as a final irrigation solution to eliminate any chemical effect on the bond strength of BS sealer to dentin-root canal walls [21,28-29]. In addition, the canal drying condition used in this study was found to be the best to achieve the highest bond strength of a BS sealer with root-dentin walls [26]. Few studies evaluated the effect of different irrigation solutions on BS sealers. Shokouhinejad et al. found that regardless of the irrigation used as a final irrigation solution, similar bond strength of EndoSequence BS sealer to root-dentin walls was reported [23].

In contrast, Gyulbenkiyan et al. reported that using chitosan-citrate (0.6%) as the final irrigation solution contributed to the most adaptation of the BS sealer/dentin interface compared to other tested solutions [21]. It is well established that the efficiency of irrigation solutions in RC debridement is enhanced by irrigation/activation methods used such as US irrigation, EndoVac, and laser compared to conventional syringe irrigation [30-34]. Indeed, our study findings support previous studies, as the SEM gap analysis results revealed that conventional syringe irrigation has the most gap size between the BC sealer and root-dentin walls compared to US and laser activation methods (Figure 2). This may be explained, at least in part, by the failure of such methods to effectively remove the smear layer and RC debris. Furthermore, it has been shown that proper adaptation and adhesion of the RC filling material, especially at the apical part of the RC system, significantly reduced microleakage and enhanced root fracture resistance [35,36]. In our study, the Nd:YAG laser activation method is shown to be superior in sealer/dentin interface adaptation presented with statistically significantly fewer gaps as evaluated via SEM compared to conventional irrigation and US activation methods. Thus, from the biological point of view, this would enhance the sealing ability of the BS sealer within the RC system and prevent bacterial leakage from outside the root via dentinal tubules from the periradicular area. As a result, we can argue that this may positively promote treatment outcomes [37-40]. Ozkocak et al. found that the bond strength of RC sealers is modulated by the different dentin surface treatments [22]. Thus, EDTA and Er:YAG laser application removed the smear layer and increased bond strength when tested in resin RC sealers and BS sealers [22]. This result is in agreement with our finding, where Nd:YAG laser activation showed a clean and smooth dentin surface under the SEM between the sealer/dentin interface, which may explain, in part, the significant increase in bond strength compared to conventional and the US tested groups.

Moreover, Esteves-Oliveira et al., in a pilot study, tested the effect of three main types of lasers on dentin RC permeability and morphology [41]. They concluded that all three types may have different yet interacting applications as an adjuvant tool to endodontic treatment, and interestingly, the Nd:YAG laser seems to decrease dentin permeability compared to Er:YAG and diode laser, where they seem to increase dentin permeability [41]. This information warrants further investigation to understand both sides’, i.e. biological and mechanical, advantages and disadvantages in the Era of endodontics. Understandably, the cost of including a laser machine in a dental clinic setting exceeds that of using conventional and even US methods in RC system irrigation; however, whether this may clinically affect treatment outcomes is a question yet worth to be answered in future clinical studies. Finally, some limitations of the present study may include, sample size, the selected morphology of the teeth, and the use of a single BC sealer brand from (TotalFill® BC Sealer™, FKG Dentaire, Switzerland) in all tested groups. This was planned to give more standardized conditions, and hence, more reliable results. However, in real clinical daily practice, treated teeth may have different roots morphology, and clinicians may use BC sealers from different branding companies in which minor composition variations may have different bonding results. All of these factors may be overcome by increasing the tested sample size. Thus, future clinical studies are needed to answer these questions. 

## Conclusions

Within the limitations of this study, the irrigation method protocol during root canal treatment, affects the adhesion and bond strength of bioceramic sealer to the root canal walls system. Further studies are warranted to investigate whether the activation of irrigation solutions with Nd:YAG laser can perform the same under complicated root canals morphology. 
